# Supplementary material for: Integration of QTL Mapping and Whole Genome Sequencing Identifies Candidate Genes for Alkalinity Tolerance in Rice (Oryza sativa)
Source: Int J Mol Sci. 2022 Oct 4;23(19):11791. doi: 10.3390/ijms231911791 (PMC9569586; doi:10.3390/ijms231911791)
Supplement: Supplementary file 1 [file ijms-23-11791-s001.zip › Table S1.pdf]

Table S1. List of additive QTLs identified under non-stress (control) condition at seedling stage by ICIM in the Cocodrie × N22 RIL population.

Tra it	QTLs	C hr	Positi on	Left Marker	Right Marker	LO D[a]	PVE (%)[b]	Additive effect	No. of genes in QTL interval	Parental allele for increased effect
SH	*qSHL1.*	1	4	S1_6583	S1_1098	2.7	3.8	1.57	144	N22
SH	*qSHL1.*	1	92	S1_2212	S1_2219	2.2	2.8	-1.90	6	Cocodrie
SH	*qSHL1.*	1	189	S1_3802	S1_3828	21.	38.0	5.03	40	N22
SH	*qSHL5.*	5	45	S5_1408	S5_1651	2.1	2.9	1.37	229	N22
SH	*qSHL8.*	8	110	S8_2601	S8_2602	5.3	7.5	2.20	3	N22
RT	*qRTL13*	1	189	S1_3802	S1_3828	3.1	7.9	0.47	40	N22
RT	*qRTL6.*	6	25	S6_4715	S6_5656	2.9	8.2	0.48	119	N22
RT	*qRTL8.*	8	125	S8_2806	S8_2831	2.0	4.7	0.36	39	N22
RS	*qRSR1.*	1	188	S1_3802	S1_3828	7.9	16.9	-0.03	40	Cocodrie
RS	*qRSR3.*	3	187	S3_3629	S3_3636	2.8	5.3	0.02	9	N22
SN	*qSNC1.*	1	4	S1_6583	S1_1098	2.7	3.8	39.36	144	N22
SN	*qSNC1.*	1	92	S1_2212	S1_2219	2.2	2.8	-47.56	6	Cocodrie
SN	*qSNC1.*	1	189	S1_3802	S1_3828	21.	38.0	125.64	40	N22
SN	*qSNC5.*	5	45	S5_1408	S5_1651	2.1	2.9	34.31	229	N22
SN	*qSNC8.*	8	110	S8_2602	S8_2601	5.3	7.5	55.11	3	N22
SK	*qSKC1.*	1	4	S1_6583	S1_1098	2.7	3.8	31.49	144	N22
SK	*qSKC1.*	1	92	S1_2212	S1_2219	2.2	2.8	-38.04	6	Cocodrie
SK	*qSKC1.*	1	189	S1_3802	S1_3828	21.	38.0	100.51	40	N22
SK	*qSKC5.*	5	45	S5_1408	S5_1651	2.1	2.9	27.49	229	N22
SK	*qSKC8.*	8	110	S8_2602	S8_2601	5.3	7.5	44.09	3	N22
RK	*qRKC1.*	1	4	S1_6583	S1_1098	2.1	3.1	31.74	144	N22
RK	*qRKC1.*	1	92	S1_2212	S1_2219	2.1	2.7	-41.78	6	Cocodrie
RK	*qRKC1.*	1	189	S1_3802	S1_3828	20.	36.5	109.67	40	N22
RK	*qRKC8.*	8	110	S8_2602	S8_2601	5.8	8.4	52.28	3	N22
RN	*qRNK4.*	4	40	S4_1661	S4_1688	3.1	8.4	0.77	26	N22
RN	*qRNK8.*	8	0	S8_2612	S8_4980	2.0	5.0	0.58	37	N22

SHL, shoot length; RTL, root length; RSR, root to shoot ratio; SNC, shoot Na^+ concentration; SKC, shoot K^+ concentration; RNC, root Na^+ concentration, RKC, root K^+ concentration; SNK, shoot Na/K ratio, RNK: root Na/K ratio.

[a]LOD, logarithm of odds

[b]PVE (%), percentage phenotypic variance explained by the QTL
