# Supplementary material for: Integration of QTL Mapping and Whole Genome Sequencing Identifies Candidate Genes for Alkalinity Tolerance in Rice (Oryza sativa)
Source: Int J Mol Sci. 2022 Oct 4;23(19):11791. doi: 10.3390/ijms231911791 (PMC9569586; doi:10.3390/ijms231911791)
Supplement: Supplementary file 1 [file ijms-23-11791-s001.zip › Table S2.pdf]

Table S2. List of additive QTLs identified by Interval Mapping (IM) under alkaline stress at seedling stage.

Trait	QTL	Chr	Position	Left Marker	Right Marker	LOD[a]	PVE (%)[b]	Additive effect	Parental allele for increasing effect
AK	*qAKT9.2*	9	82	S9_2051925	S9_2048044	4.4	10.6	0.42	N22
CH	*qCHL1.3*	1	172	S1_3413237	S1_3549627	2.7	5.6	-1.07	Cocodrie
CH	*qCHL1.3*	1	185	S1_3756187	S1_3774070	3.0	5.9	1.10	N22
CH	*qCHL9.2*	9	83	S9_2049153	S9_2074834	6.9	12.9	-1.63	Cocodrie
CH	*qCHL9.2*	9	98	S9_2238577	S9_2250418	3.1	5.9	-1.09	Cocodrie
SH	*qSHL1.05*	1	29	S1_5121056	S1_5498898	3.5	4.0	1.37	N22
SH	*qSHL1.05*	1	33	S1_5920937	S1_6222811	3.7	4.5	1.44	N22
SH	*qSHL1.37*	1	182	S1_3708929	S1_3727318	16.3	16.8	2.83	N22
SH	*qSHL1.38*	1	189	S1_3802368	S1_3828677	23.2	21.6	3.19	N22
SH	*qSHL3.13*	3	78	S3_1386089	S3_1394855	2.7	3.2	-1.22	Cocodrie
SH	*qSHL8.17*	8	58	S8_1733825	S8_1744356	2.7	3.3	-1.23	Cocodrie
RT	*qRTL3.28*	3	134	S3_2838670	S3_2844133	2.7	6.8	0.49	N22
RS	*qRSR1.37*	1	182	S1_3708929	S1_3727318	9.2	12.2	-0.05	Cocodrie
RS	*qRSR3.14*	3	82	S3_1451360	S3_1472484	3.9	5.6	0.03	N22
RS	*qRSR8.16*	8	57	S8_1676929	S8_1681290	2.7	3.9	0.03	N22
SN	*qSNC8.0*	8	0	S8_261276	S8_498009	2.6	6.6	87.31	N22
SN	*qSNC9.2*	9	82	S9_2051925	S9_2048044	4.2	10.2	109.39	N22
SN	*qSNC12.*	12	68	S12_199683	S12_203757	2.7	6.7	-89.85	Cocodrie
RN	*qRNC8.0*	8	0	S8_261276	S8_498009	2.9	7.1	97.25	N22
RN	*qRNC9.2*	9	82	S9_2051925	S9_2048044	4.0	9.9	115.08	N22
RN	*qRNC12.*	12	68	S12_199683	S12_203757	2.9	7.2	-99.89	Cocodrie
SK	*qSKC8.0*	8	0	S8_261276	S8_498009	2.7	8.1	-20.32	Cocodrie
SK	*qSKC9.2*	9	82	S9_2051925	S9_2048044	3.7	11.1	-23.91	Cocodrie
RK	*qRKC8.0*	8	0	S8_261276	S8_498009	2.9	8.7	-20.50	Cocodrie
RK	*qRKC9.2*	9	82	S9_2051925	S9_2048044	4.0	11.9	-24.17	Cocodrie
SN	*qSNK4.1*	4	40	S4_1661217	S4_1688078	2.9	7.4	0.66	N22
SN	*qSNK9.1*	9	76	S9_1925114	S9_1928815	2.6	6.5	0.62	N22
RN	*qRNK4.1*	4	41	S4_1679521	S4_1783816	3.2	7.5	0.83	N22
RN	*qRNK9.1*	9	76	S9_1925114	S9_1928815	2.7	6.3	0.76	N22

AKT, alkalinity tolerance score; CHL, chlorophyll content; SHL, shoot length; RTL, root length; RSR, root to shoot ratio; SNC, shoot Na^+ concentration; SKC, shoot K^+ concentration; RNC, root Na^+ concentration; RKC, root K^+ concentration; SNK, shoot Na/K ratio; RNK, root Na/K ratio.

[a]LOD, logarithm of odds

[b]PVE (%), percentage phenotypic variance explained by the QTL
