# Supplementary material for: Integration of QTL Mapping and Whole Genome Sequencing Identifies Candidate Genes for Alkalinity Tolerance in Rice (Oryza sativa)
Source: Int J Mol Sci. 2022 Oct 4;23(19):11791. doi: 10.3390/ijms231911791 (PMC9569586; doi:10.3390/ijms231911791)
Supplement: Supplementary file 1 [file ijms-23-11791-s001.zip › Table S3.pdf]

**Table S3.** List of additive QTLs identified by Interval Mapping (IM) under non-stress (control) condition in the Cocodrie/N22 RIL population.

| Trait | QTLs | Chr | Position | Left Marker | Right Marker | LOD[a] | PVE (%)[b] | Additive effect | Parental allele for Increasing effect |
|---|---|---|---|---|---|---|---|---|---|
| SH | *qSHL1.0* | 1 | 33 | S1_592093 | S1_622281 | 3.4 | 5.7 | 2.34 | N22 |
| SH | *qSHL1.2* | 1 | 92 | S1_221280 | S1_221915 | 2.8 | 4.6 | -2.94 | Cocodrie |
| SH | *qSHL1.3* | 1 | 168 | S1_332399 | S1_334150 | 3.6 | 5.9 | 2.38 | N22 |
| SH | *qSHL1.3* | 1 | 189 | S1_380236 | S1_382867 | 17.8 | 24.9 | 4.94 | N22 |
| SH | *qSHL8.1* | 8 | 64 | S8_181736 | S8_185491 | 3.1 | 5.1 | -2.22 | Cocodrie |
| SH | *qSHL8.2* | 8 | 107 | S8_254677 | S8_255010 | 3.2 | 5.2 | 2.23 | N22 |
| RT | *qRTL1.3* | 1 | 189 | S1_380236 | S1_382867 | 3.9 | 7.7 | 0.55 | N22 |
| RT | *qRTL6.0* | 6 | 25 | S6_471528 | S6_565644 | 3.8 | 8.2 | 0.57 | N22 |
| RS | *qRSR1.3* | 1 | 188 | S1_380236 | S1_382867 | 7.5 | 15.5 | -0.03 | Cocodrie |
| RS | *qRSR3.1* | 3 | 78 | S3_138608 | S3_139485 | 2.9 | 5.9 | 0.02 | N22 |
| SN | *qSNC1.0* | 1 | 33 | S1_592093 | S1_622281 | 3.4 | 5.7 | 58.45 | N22 |
| SN | *qSNC1.2* | 1 | 92 | S1_221280 | S1_221915 | 2.8 | 4.6 | -73.54 | Cocodrie |
| SN | *qSNC1.3* | 1 | 189 | S1_380236 | S1_382867 | 17.8 | 24.9 | 123.49 | N22 |
| SN | *qSNC8.1* | 8 | 64 | S8_181736 | S8_185491 | 3.1 | 5.1 | -55.43 | Cocodrie |
| SN | *qSNC8.2* | 8 | 107 | S8_254677 | S8_255010 | 3.2 | 5.2 | 55.66 | N22 |
| SK | *qSKC1.0* | 1 | 33 | S1_592093 | S1_622281 | 3.4 | 5.7 | 46.76 | N22 |
| SK | *qSKC1.2* | 1 | 92 | S1_221280 | S1_221915 | 2.8 | 4.6 | -58.83 | Cocodrie |
| SK | *qSKC1.3* | 1 | 168 | S1_332399 | S1_334150 | 3.6 | 5.9 | 47.53 | N22 |
| SK | *qSKC1.3* | 1 | 189 | S1_380236 | S1_382867 | 17.8 | 24.9 | 98.79 | N22 |
| SK | *qSKC8.1* | 8 | 64 | S8_181736 | S8_185491 | 3.1 | 5.1 | -44.34 | Cocodrie |
| SK | *qSKC8.2* | 8 | 107 | S8_254677 | S8_255010 | 3.2 | 5.2 | 44.53 | N22 |
| RK | *qRKC1.0* | 1 | 33 | S1_592093 | S1_622281 | 3.0 | 5.7 | 49.52 | N22 |
| RK | *qRKC1.2* | 1 | 92 | S1_221280 | S1_221915 | 2.7 | 4.9 | -64.49 | Cocodrie |
| RK | *qRKC1.3* | 1 | 189 | S1_380236 | S1_382867 | 16.6 | 26.5 | 107.68 | N22 |
| RK | *qRKC8.1* | 8 | 64 | S8_181736 | S8_185491 | 2.8 | 5.2 | -47.13 | Cocodrie |
| RK | *qRKC8.2* | 8 | 107 | S8_254677 | S8_255010 | 3.5 | 6.4 | 52.67 | N22 |
| RN | *qRNK4.1* | 4 | 40 | S4_166121 | S4_168807 | 3.2 | 6.9 | 0.83 | N22 |
| RN | *qRNK12.* | 12 | 68 | S12_19968 | S12_20375 | 2.7 | 5.7 | 0.76 | N22 |

SHL, shoot length; RTL, root length; RSR, root to shoot ratio; SNC, shoot $Na^+$ concentration; SKC, shoot $K^+$ concentration; RNC, root $Na^+$ concentration; RKC, root $K^+$ concentration; SNK, shoot Na/K ratio; RNK, root Na/K ratio.

[a]LOD, logarithm of odds

[b]PVE (%), percentage phenotypic variance explained by the QTL
